# Fabrication of Hollow Structures in Photodegradable Hydrogels Using a Multi-Photon Excitation Process for Blood Vessel Tissue Engineering

**DOI:** 10.3390/mi11070679

**Published:** 2020-07-13

**Authors:** Uran Watanabe, Shinji Sugiura, Masayuki Kakehata, Fumiki Yanagawa, Toshiyuki Takagi, Kimio Sumaru, Taku Satoh, Masato Tamura, Yoichiroh Hosokawa, Kenji Torizuka, Toshiyuki Kanamori

**Affiliations:** 1 Biotechnology Research Institute for Drug Discovery, National Institute of Advanced Industrial Science and Technology (AIST), Tsukuba, Ibaraki 305-8565, Japan; s1310743@gmail.com (U.W.); fumikiyanagawa@gmail.com (F.Y.); t.takagi@aist.go.jp (T.T.); k.sumaru@aist.go.jp (K.S.); taku.satoh@scetra.or.jp (T.S.); masato.tamura@rcnp.osaka-u.ac.jp (M.T.); t.kanamori@aist.go.jp (T.K.); 2 School of Integrative and Global Majors, University of Tsukuba, Tsukuba, Ibaraki 305-8577, Japan; 3 Cellular and Molecular Biotechnology Research Institute, National Institute of Advanced Industrial Science and Technology (AIST), Tsukuba, Ibaraki 305-8565, Japan; 4 Research Institute for Advanced Electronics and Photonics, National Institute of Advanced Industrial Science and Technology (AIST), Tsukuba, Ibaraki 305-8568, Japan; kakehata-masayuki@aist.go.jp (M.K.); k.torizuka@aist.go.jp (K.T.); 5 Division of Materials Science, Nara Institute of Science and Technology, Ikoma, Nara 630-0192, Japan; hosokawa@ms.naist.jp

**Keywords:** hydrogel, microfabrication, microfluidics, tissue engineering, multi-photon excitation

## Abstract

Engineered blood vessels generally recapitulate vascular function in vitro and can be utilized in drug discovery as a novel microphysiological system. Recently, various methods to fabricate vascular models in hydrogels have been reported to study the blood vessel functions *in vitro*; however, in general, it is difficult to fabricate hollow structures with a designed size and structure with a tens of micrometers scale for blood vessel tissue engineering. This study reports a method to fabricate the hollow structures in photodegradable hydrogels prepared in a microfluidic device. An infrared femtosecond pulsed laser, employed to induce photodegradation via multi-photon excitation, was scanned in the hydrogel in a program-controlled manner for fabricating the designed hollow structures. The photodegradable hydrogel was prepared by a crosslinking reaction between an azide-modified gelatin solution and a dibenzocyclooctyl-terminated photocleavable tetra-arm polyethylene glycol crosslinker solution. After assessing the composition of the photodegradable hydrogel in terms of swelling and cell adhesion, the hydrogel prepared in the microfluidic device was processed by laser scanning to fabricate linear and branched hollow structures present in it. We introduced a microsphere suspension into the fabricated structure in photodegradable hydrogels, and confirmed the fabrication of perfusable hollow structures of designed patterns via the multi-photon excitation process.

## 1. Introduction

In recent years, the cost of drug development has increased exponentially [1], and the success rate of clinical trials has been decreasing every year [2]. One of the reasons for this is that the results of animal experiments cannot be directly extrapolated to clinical trials due to the difference in species between animals and humans. Under these circumstances, expectations for in vitro assays using cultured cells of human origin are increasing, and in particular, microphysiological systems (MPS), which are biomimetic devices using microfabrication technology, are receiving immense attention. 

Blood vessels not only carry oxygen and nutrients in the body [3], but are also greatly involved in angiogenesis and invasion of cancer [4]. Moreover, they are related to drug delivery and pharmacokinetics, and therefore engineered blood vessels are assumed to be used in drug discovery as a novel MPS. Traditionally, two-dimensional (2D) culture models have been used to characterize the shear stress response of endothelial cells [5,6,7]; however, 2D cell culture systems generally lose cellular functions due to the lack of a biological environment, such as three-dimensionally ordered cell–cell interactions, cell–extracellular matrix interactions, and signaling mechanisms [8]. Therefore, three-dimensional (3D) vascular models are desirable to recapitulate vascular functions in vivo [9,10,11]. Furthermore, it is necessary to evaluate multiple compounds at different concentrations for drug development, and therefore a multithroughput format, ideally an array format, of the 3D vascular model is required for use in drug discovery.

Another aspect of in vitro culture of 3D vascular models is their medium flow. The vascular model is ideally required to be cultured under shear stress generated by medium flow to maintain the blood vessel functions [9,12,13]. Microfluidic devices are useful tools for medium perfusion, and have recently gained immense attentions as an “organ-on-a chip” exhibiting organ level functions in vitro [14]. In the microfluidic device, vascular endothelial cells were cultured under the controlled shear stress generated by medium perfusion [15,16]. The flow rate can be controlled precisely even over a logarithmic range by designing the microfluidic channel network structure [17,18]. In addition, microfluidic devices can provide a physiological culture environment controlled in the cellular length scale [19]. Perfusable and functional microvascular networks in a 3D extracellular matrix in microfluidic devices have been formed by vasculogenesis [9,10]. However, in this case, controlling the size and structure of the microvascular network is generally difficult because vasculogenesis is a spontaneous phenomenon. Therefore, fabrication technology for an array-formatted 3D vascular model with controlled size is important to realize the application of 3D vascular model models for drug discovery.

Recently, fabrication methods for 3D vascular models have been developed for mimicking the in vivo cell behavior [20,21]. Methods are available to fabricate the vascular network structures in hydrogels using water-soluble filaments and biodegradable elastomer fabricated by 3D printing [22], and 3D stamping [23] techniques, respectively. Moreover, one method fabricates the microvascular-sized fluidic channels in a hydrogel formed by the molding techniques using a silicon elastomer [24]. In these methods, the vascular models were constructed by introducing vascular endothelial cells into the fabricated structures; however, it is generally difficult to control the blood vessel structure to tens of micrometers in these hydrogels. 

Photofabrication technology is advantageous for fabricating micrometer-sized 3D structures in hydrogels for tissue engineering [25,26]. To date, the stereolithography method [27,28], photo-degradation method [29,30] and laser-induced degradation method [31] have been used for the photofabrication of hydrogels. Laser-induced degradation is a powerful technique to fabricate hollow structures in various biomaterials including collagen [32], silk protein [33], covalently crosslinked poly(ethylene glycol) (PEG) [34,35], and PEGylated fibrinogen hydrogels [36]. Photo-degradation of photodegradable hydrogels via multi-photon excitation is another technique for fabricating the hollow structures in photodegradable hydrogels [37,38,39]. PEG-based photodegradable hydrogel encapsulating cells were locally degraded using a two-photon laser-scanning microscope and arbitrary 3D structures were created within the hydrogel. Perfusable structures were fabricated in hydrogels using these techniques [32,34,35]. Several studies have reported the culturing of endothelial cells in the fabricated structures for blood vessel tissue engineering [32,35,40]. Medium perfusion is important for supplying nutrients and applying shear stress to the endothelial cells for blood vessel tissue engineering. In order to perfuse the medium into the fabricated hollow structure, the microfluidic device acts as a powerful tool. To the best of our knowledge, no report is available on the fabrication of the perfusable hollow structure of the photodegradable hydrogel for blood vessel tissue engineering.

In the present study, we formed photodegradable hydrogels in a perfusion culture microfluidic device, and fabricated hollow structures with designed sizes and shapes in the hydrogels via multi-photon excitation process by scanning an infrared femtosecond pulsed laser. The microfluidic device was placed on the sample stage in the microscope, on which the laser was introduced. The X-Y-Z position of the sample stage was controlled by the program, which enabled us to scan the designed 3D structure with micrometer-scale precision in the hydrogels. We examined the hydrogel composition to maintain the fabricated structure and cell adhesion. We fabricated a hollow structure with a scale of tens of micrometers in the photodegradable hydrogels of the microfluidic device, and confirmed the fabrication of hollow structures by perfusing a suspension of microbeads into the fabricated structures.

## 2. Materials and Methods

### 2.1. Synthesis of Azide-Gelatin and Photocleabable Crosslinker

The photodegradable hydrogels were prepared by the reaction between azide-modified gelatin (azide-gelatin) and dibenzocyclooctyl-terminated photocleavable tetra-arm polyethylene glycol (DBCO-PC-4armPEG) crosslinker, as previously reported [41]. Azide-gelatin is a gelatin derivative, in which the amino moieties are modified into azide moieties by 15-azide-4,7,10,13-tetraoxapentadecanoic acid *N*-succinimidyl ester (azide-PEG_4_-NHS). Azide moieties react with DBCO moieties in DBCO-PC-4armPEG to form covalent crosslinks, thereby resulting in the formation of photodegradable hydrogels. *o*-Nitrobenzyl moieties in DBCO-PC-4armPEG are cleaved via ultraviolet irradiation, resulting in degradation of the hydrogels. 

Azide-gelatins with 15%, 24%, 50%, and 75% modification rate, which indicate the molar fraction of azide-PEG_4_-NHS ester added during synthesis to amino moieties in gelatin, were synthesized by mixing gelatin (G6144-100G, Sigma-Aldrich Co., Ltd., St. Louis, MO, USA) with azide-PEG_4_-NHS (Click Chemistry Tools Int., Inc, Scottsdale, AZ, USA) in accordance with our previous study [41], and hereafter, are denoted as Azide-gelatin (15), (24), (50), and (75), respectively. Gelatin (1.0 g) was dissolved in 50 mL dimethyl sulfoxide (DMSO, Kishida Chemical Co., Ltd., Osaka, Japan) at 60 °C. Thereafter, 44.6, 72.4, 147.5, and 221.5 mg of azide-PEG_4_-NHS were dissolved in 10 mL DMSO at 37 °C for azide-gelatin (15), (24), (50), and (75), respectively. Subsequently, the azide-PEG_4_-NHS solutions were added to the gelatin solutions, and then mixed for 2 h at 37 °C. The reaction mixtures were dialyzed to reverse osmosis water using dialysis membranes with 6–8 kD of molecular weight cut off (Spectrum Laboratories, Inc., Rancho Dominguez, CA, USA) for 2 days at 1:20 dilution after 0.5, 2, 16.5, 24.5, and 41 h. After dialysis, the reaction mixtures were frozen and lyophilized for 3 days in a freeze dryer (FDS-1000, Tokyo Rikakikai Co., Ltd., Tokyo, Japan). The molar ratio of the azide moiety to the primary amino groups in gelatin was estimated via fluorescamine assay [42] as 35%, 50%, 83%, and 94% for azide-gelatin (15), (24), (50), and (75), respectively. 

DBCO-PC-4armPEG crosslinker (MW: 13,753) was synthesized from NHS-PC-4armPEG and DBCO-PEG_4_-amine in our previous study [41].

### 2.2. Size Change of Photodegradable Hydrogels

The size change of the photodegradable hydrogel was examined using cylindrical-shaped hydrogels with different concentrations and modification rates of azide-gelatins (Table 1). The concentration of the DBCO-PC-4armPEG crosslinker was designed as 1:1 stoichiometric ratio of the charged amount of azide-PEG_4_-NHS during the synthesis of azide-gelatins and DBCO moieties in DBCO-PC-4armPEG. Azide-gelatins and DBCO-PC-4armPEG were dissolved in phosphate buffered saline (PBS, FUJIFILM Wako Pure Chemical Corp., Osaka, Japan) to prepare photodegradable hydrogels. These solutions were mixed at 1:1 volume ratio at room temperature to prepare the pregel solutions. Thereafter, 3.5 µL of the pre-gel solutions were introduced into a silicon tubes with 1.5 mm inner diameter (As One Corp., Osaka, Japan). After incubation for 3 h at room temperature for hydrogel formation, each photodegradable hydrogel was retrieved from the silicon tube. The hydrogels were incubated in PBS in 35 mm dishes for 7 days at 37 °C in a humidified incubator, and short axial lengths were measured on Days 0, 1, 4, and 7 using a microscope (CKX41, Olympus Corp., Tokyo, Japan). 

### 2.3. Cell Adhesion and Growth on Photodegradable Hydrogels

Human umbilical vein endothelial cells (HUVECs) were obtained from Lifeline Cell Technology, and were maintained in EGM-2 (Lonza Ltd., Basel, Switzerland) supplemented with 100 units/mL penicillin and 100 µg/mL streptomycin at 37 °C in 5% CO_2_ atmosphere. 

HUVEC adhesion and growth on the photodegradable hydrogels with different compositions (Table 1) were assessed using hydrogels formed in a custom-designed 8 × 4 well plate, in which a circular step structure with 6 mm diameter and 334 µm depth was fabricated in each well [43]. Next, 11 µL of the pregel solutions prepared as aforementioned, were dropped into the step structures inside the well plate. After incubation for 3 h at room temperature under humidified conditions for hydrogel formation, the hydrogels were washed with 500 µL of EGM-2. Thereafter 200 µL of 9.4 × 10^4^ cells/mL HUVEC suspension was added to the hydrogel. After the incubation for 4 h at 37 °C for cell adhesion, 300 µL of EGM-2 was added onto the hydrogels, and incubated for 7 days at 37 °C in 5% CO_2_ atmosphere. The LIVE/DEAD (Thermo Fisher Scientific Inc., Waltham, MA, USA) assay was performed based on the information provided by the company. Briefly, the hydrogels were washed twice with 500 µL of PBS, and then, incubated for 30 min at 37 °C in 200 µL of LIVE/DEAD staining solution. The bright field and fluorescent images of the stained cells on the hydrogels were acquired using an inverted fluorescent microscope (IX71, Olympus Corp., Tokyo, Japan).

### 2.4. Fabrication of Microfluidic Device

**Figure 1:**
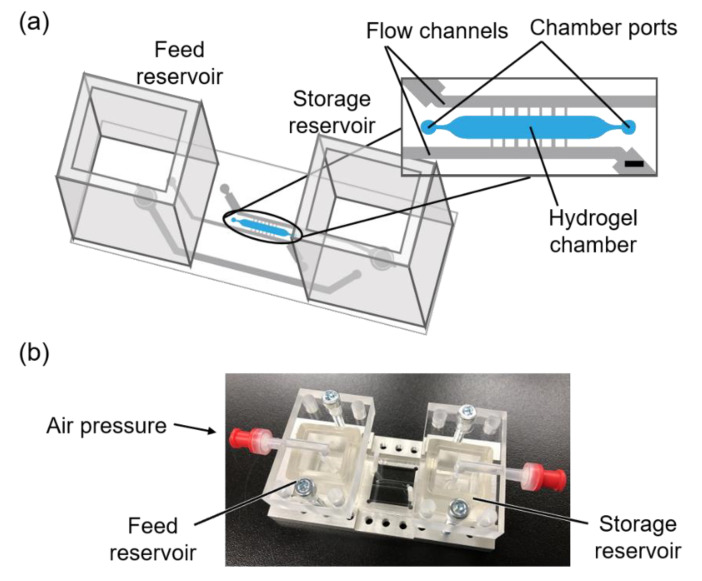
Schematic of the microfluidic device (**a**) and photograph of the fabricated microfluidic device (**b**). Scale bar: 1 mm (**a**).

We designed a microfluidic device with a hydrogel chamber, where a photodegradable hydrogel was formed, with flow channels and, feed and storage reservoirs (Figure 1). The photomask patterns were designed using an Illustrator (Adobe, CS4). The microfluidic device was fabricated by soft lithography [44,45] with a silicon master template using negative photoresist SU-8 (Nippon Kayaku Co., Ltd., Tokyo, Japan). The template comprised a multi-later photoresist pattern, and was fabricated in accordance with the manufacturer’s manual. The first layer for the hydrogel chamber was fabricated using SU-8 2075 with a resulting depth of 300 µm. The second layer for the fluid channel was fabricated using SU-8 2075 over the hydrogel chamber layer with a resulting depth of 375 µm. After the photolithography process, the silicon wafer was developed in ethyl lactate, and treated with tridecafluoro-1,1,2,2-tetrahydrooctyl-1-trichlorosilane. 

Poly (dimethylsiloxane) (PDMS, Dow Corning, Midland, MI, USA) pre-polymer and curing agent were mixed (ratio: 10:1) appropriately, poured onto the master template, and then cured for 2 h at 80 °C. Subsequently, the PDMS mold was peeled from the master template, and punctured to create chamber ports, inlet and outlet holes. The PDMS mold was bonded with a glass slide and two PDMS reservoirs by oxygen plasma treatment using a plasma reactor (PR500, Yamato Scientific Co., Ltd., Tokyo, Japan). 

### 2.5. Setup of Laser Scanning

**Figure 2:**
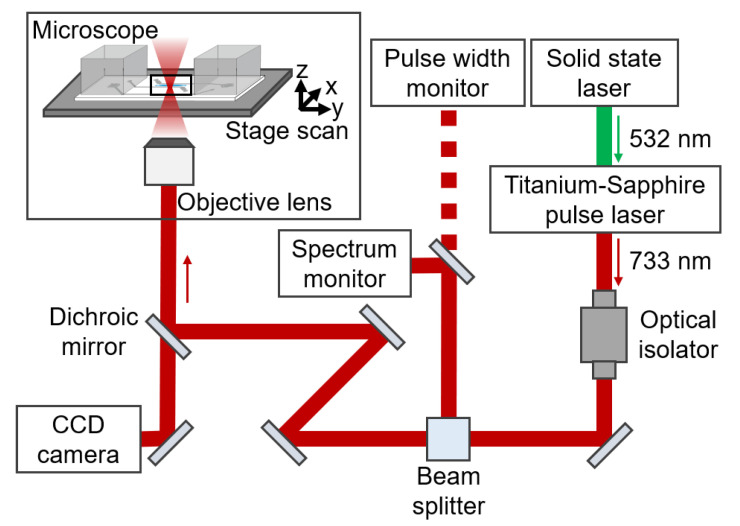
Experimental setup for laser scanning. The experimental setup comprised a solid-state laser, titanium-sapphire pulse laser, optical isolator, CCD camera, inverted microscope, and optical elements including mirrors, lenses, and monitors.

The experimental apparatus for the laser scanning is schematically illustrated in Figure 2. Laser scanning was carried out using a Titanium-Sapphire pulsed laser (Tsunami, Spectra Physics, Inc., Mountain View, CA, USA) coupled to an inverted microscope (IX51, Olympus Corp. Tokyo, Japan), equipped with step motors (KXG06020-GA, Suruga Seiki Corp., Shizuoka, Japan) for scanning the sample stage. A 532 nm solid-state laser (Millennia XJ, Spectra Physics, Inc., Mountain View, CA, USA) with 7-W power was supplied to the Tsunami. The Tsunami generated 80-MHz laser pulse trains, 733 nm center wavelength, and 8 nm full-width half-maximum, corresponding to a ~100 fs transform limited pulse. The pulse spectrum was continuously monitored by a spectrometer (CCS175, Thorlabs, Inc., Newton, NJ, USA), and the laser power directed to the microscope was measured using a power meter (30A-SH-V1, Ophir Optronics, Ltd., Danvers MA, USA). The pulse width before the objective lens measured by an auto-correlator (Model 409, Spectra Physics Inc., Mountain View, CA, USA) was 230 fs. The pulse became longer than the transform-limited pulse due to dispersion of the optical isolator, which was placed between the Tsunami and the microscope to reject the reflection from the microscopes to the laser. This laser with 250 mW average power was introduced into the photodegradable hydrogels at the sample stage through a ×10 objective lens (NA = 0.4, Olympus Corp., Tokyo, Japan). The sample stage was scanned using LabVIEW software (LabVIEW 16.0).

### 2.6. Fabrication of a Hollow Structure in a Photodegradable Hydrogel in the Microfluidic Device

The pregel solution was introduced into the hydrogel chamber from a hydrogel port in the microfluidic device and gelled for 3 h at room temperature in the dark. The next day, hollow structures were fabricated using stage scanning controlled by LabVIEW software. The sample stage was moved in the direction of X axial at a scanning velocity of V_X_. In order to fabricate continuous hollow structures, laser scanning was repeated in a stepwise manner by moving the sample stage in the direction of Y and Z axial with an average interval of ΔY and ΔZ, respectively. The typical laser scanning condition was a V_X_ of 750 µm/s, ΔY of 0.7 µm, and ΔZ of 1.0 µm. We fabricated linear and branched hollow structures in the hydrogel.

### 2.7. Perfusion of Buffer in the Hollow Structure Fabricated in the Photodegradable Hydrogel

Most of the previously reported MPS use a syringe pump [46,47] outside the chip or a peristaltic pump [48,49,50] to deliver the culture solution. As such configuration requires complicated tube connections, it is difficult to achieve high culture throughput MPS. To solve these issues, the authors have recently developed a pressure-driven multithroughput MPS [16,51,52]. The pressure-driven MPS allows the culture solution and cells to be added to and collected from the chip by opening the lid, and further permitting medium circulation by attaching the lid and fixing it to a dedicated pneumatic attachment. Therefore, pressure-driven MPS is applicable for constructing a multithroughput system, and is therefore, suitable for use in drug discovery. 

We adopted pressure-driven flow for buffer perfusion after fabricating the hollow structure in the photodegradable hydrogel [16,51,52]. After laser scanning, PBS was added to the feed reservoir and the microfluidic device was connected to the pressure control system (ASTF0301, Engineering System Co., Ltd., Matsumoto, Japan) for pressure-driven perfusion ([16,52]. Humidified air with 5% CO_2_ was introduced into a sequence pressure control system via an electromagnetic air pump (MV-6005VP, Enomoto Micro Pump Mfg. Co., Ltd., Tokyo, Japan), and a pressure of 20 kPa was applied to the feed reservoir until the degraded polymer was flushed away. The incubator was maintained at 37 °C. 

Suspension of red fluorescent polymer microspheres with 0.023 µm diameter (R25, Thermo Fisher Scientific, Inc., Waltham, MA, USA) was mixed with PBS at the volume ratio of 1:3. Two days after scanning, the microsphere suspension was added into the feed reservoir and flowed into the fabricated structures by applying a pressure of 5 kPa for 1 and 3 min for the liner and branched hollow structures, respectively, to confirm their fabrication.

## 3. Results

### 3.1. Size Change of Photodegradable Hydrogels

**Figure 3:**
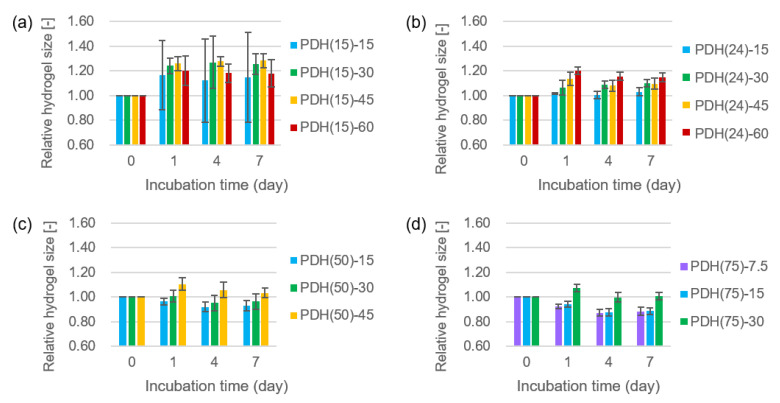
Relative size change of the photodegradable hydrogels with different compositions for 7 days. The hydrogels with compositions listed in Table 1 were prepared with azide-gelatin(15) (**a**), azide-gelatin(24) (**b**), azide-gelatin(50) (**c**), and azide-gelatin(75) (**d**). Error bars represent standard deviations of the relative hydrogel size (*n* = 4).

The photodegradable hydrogels used in this study were comprised of gelatin, a natural polymer with biocompatible and cell adhesive properties [53]; however, swelling limited their applications into tissue engineering [54,55]. In particular, for the fabrication of micrometer sized hollow structures, size change of the hydrogels disrupts the shape of the fabricated structures. We explored the composition of the photodegradable hydrogels that were resistant to size change (Table 2, Figure 3). In the case of hydrogels prepared using azide-gelatin (15), the size of all hydrogels increased after 7 days of incubation. Moreover, some PDH(15)-15 and PDH(15)-30 did not maintain their original shape at Day 7 due to their softness after swelling. In the case of hydrogels prepared using azide-gelatin (24), (50), and (75), the higher concentration of azide gelatin extensively increased the hydrogel size. In contrast, PDH(50)-15, PDH(50)-30, PDH(75)-7.5, and PDH(75)-15 exhibited shrinkage during 7 days of incubation. Remarkably, PDH(24)-15, PDH(50)-30, PDH(50)-45, and PDH(75)-30 exhibited size changes of less than 5% of their original size, and were indicated as appropriate compositions for maintaining the hydrogel size and shape. 

### 3.2. HUVEC Adhesion and Growth on Photodegradable Hydrogels 

**Figure 4:**
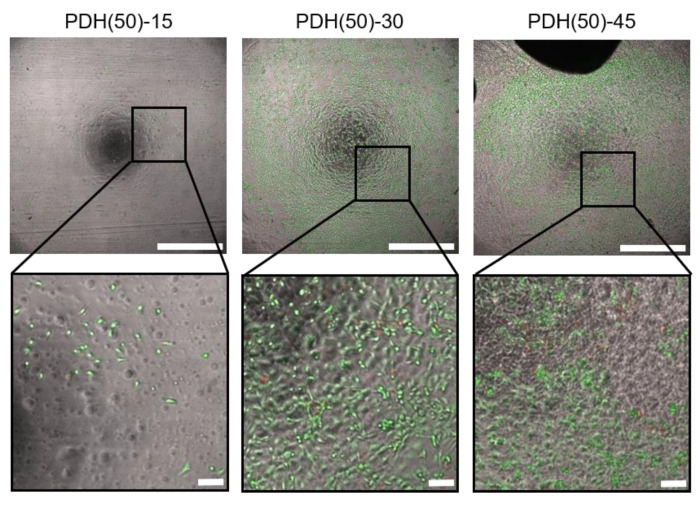
Image of HUVECs cultured on photodegradable hydrogels prepared with Azide-gelatin(50). Scale bar: 1 mm in the left panel and 0.1 mm in the right panel. The bright field and fluorescent images of the cells on the hydrogels were acquired and merged after LIVE/DEAD staining. Green and red colors indicate live and dead cells, respectively. HUVECs adhered and proliferated partially on PDH(50)-15, uniformly on PDH(50)-30, and nonuniformly on PDH(50)-45.

In order to explore the composition of photodegradable hydrogels appropriate for HUVEC adhesion and growth, HUVECs were cultured for 7 days on photodegradable hydrogels with different compositions (Table 2). Figure 4 depicts the images of HUVECs on the photodegradable hydrogels prepared using azide-gelatin (50) after LIVE/DEAD staining as representative examples. Few dead cells were observed on all hydrogels tested (Table 2). HUVECs adhered and proliferated on the photodegradable hydrogels prepared from azide-gelatin (15) and (24); however, adhesion and proliferation were nonuniform at azide-gelatin concentrations higher than 45 mg/mL. Moreover, HUVECs also adhered and proliferated on the photodegradable hydrogels prepared from Azide-gelatin (50) and (75); however, adhesion and proliferation were nonuniform at azide-gelatin concentrations higher than 45 mg/mL, and limited partial growth was observed at azide-gelatin concentrations lower than 15 mg/mL. 

### 3.3. Fabrication of Hollow Structure Using Multi-Photon Excitation Process

Based on the results of size change and HUVEC adhesion and growth, PDH(50)-30 was used for the fabrication of the hollow structures. The photodegradable hydrogels were formed in the microfluidic device and processed by laser scanning at *V*_X_ of 750 µm/s, *ΔY* of 0.7 µm, and *ΔZ* of 1.0 µm. In the preliminary experiment, we tried different scanning conditions. In general, the faster *V*_X_, the wider *ΔY* and *ΔZ* lead to insufficient degradation for perfusion, and we observed stable fabrication of hollow structures in the scanning condition of *V*_X_ of 750 µm/s, *ΔY* of 0.7 µm, and *ΔZ* of 1.0 µm. We fabricated linear and branched hollow structures with dimensions of 25 µm × 25 µm in the Y × Z cross-section. It took 1.3 and 2.7 h of laser scanning for fabricating the linear and branched hollow structures, respectively. The fabricated structures had a yellowish color immediately after laser scanning indicating that the *o*-nitrobenzyl moieties in the DBCO-PC-4armPEG crosslinker was cleaved by ultraviolet irradiation. The degraded polymer was flushed away within a day by applying a pressure of 20 kPa. We also tried a larger hollow structure of 50 µm × 50 µm in the Y × Z cross-section. It took 4.5 h of laser scanning to fabricate a linear hollow structure. This structure is at the larger limit of our present experiment in terms of realistic working time provided by our research environment.

**Figure 5:**
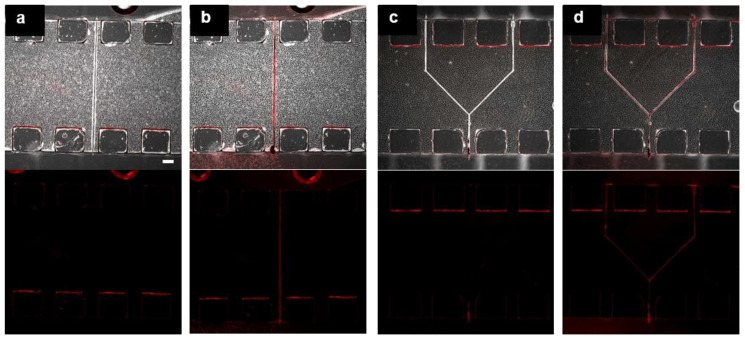
Fabricated hollow structures in a photodegradable hydrogel prepared in the microfluidic device. Merged (top) and fluorescence (bottom) images of the linear (**a**,**b**) and branched (**c**,**d**) structures before (**a**,**c**) and after (**b**,**d**) introduction of microsphere suspension. (Scale bar: 200 µm.).

We confirmed the fabrication of perfusable hollow structures by introducing a microsphere suspension. The red microspheres (diameter: 0.023 µm) were observed in the fabricated linear and branched hollow structures and flow channels (Figure 5). The structures maintained their shape for at least 2 days without hydrogel swelling. Moreover, we tried to introduce microspheres with 9.9 µm diameter into the hollow structure with 25 µm × 25 µm in the Y × Z cross-section. However, we observed multiple stacked microspheres in the hollow structure.

## 4. Discussion

We have established at method to fabricate perfusable hollow structures in photodegradable hydrogels of a microfluidic device. The infrared femtosecond pulsed laser-induced photodegradation of the hydrogels via multi-photon excitation process. Our method enabled us to fabricate hollow structures as small as 25 µm × 25 µm cross sections, whereas structures smaller than 100 µm were fabricated using 3D printing [22], 3D stamping technique [23], and molding technique [24]. Furthermore, we designed a scanning program for fabricating linear and branched hollow structures, and the corresponding hollow structures were fabricated as programmed. Therefore, our method is applicable for fabrication of various structures, including arrays of hollow structures, in photodegradable hydrogels. 

In a previous study, perfusable hollow structures were fabricated in the hydrogel using photodegradation techniques [32,34,35], and then endothelial cells were cultured in those fabricated structures [32,35,40]. Compared to these studies, our study’s differentiating characteristic was the fabrication of hollow structures in microfluidic devices, which enabled us to perfuse the medium through these fabricated hollow structures. The pressure-driven flow of the buffer was applied to these structures in the photodegradable hydrogel. Most of the previously reported MPS uses a syringe pump [46,47] outside the chip or a peristaltic pump [48,49,50] to deliver the culture solution. As such a configuration requires complicated tube connections, it is difficult to achieve high culture throughput MPS. To solve these issues, the authors have recently developed a pressure-driven multithroughput MPS [16,51,52]. The pressure-driven MPS allows the culture solution and cells to be added to and collected from the chip by opening the lid, and medium circulation by attaching the lid and attaching it to a dedicated pneumatic attachment. In addition, pressure-driven MPS is applicable to the construction of a multithroughput system, and is therefore suitable for use in drug discovery. Therefore, the method to fabricate hollow structures in pressure-driven microfluidic devices is potentially useful for multithroughput culture of engineered vascular models.

Maintaining the hydrogel’s shape and size is a key factor in influencing tissue engineering. In general, hydrogels swell or shrink after preparation. Our photodegradable hydrogel was prepared via a crosslinking reaction between an azide-gelatin and a DBCO-PC-4armPEG crosslinker. The hydrogel is comprised of chemical components with different characteristics, including azide moieties, gelatin, DBCO moieties, *o*-nitrobenzyl moieties, and PEG. Therefore, we could control the swelling and shrinkage by changing the hydrogel composition. In addition to size, cell adhesion behavior can also be controlled by changing the hydrogel make up. The importance of cell adhesion can be seen in its role in blood tissue engineering.

While examining the hydrogel composition to maintain hydrogel size, we found that the hydrogel composition for size change was less than 5% during 7 days of incubation. We observed the general tendency that higher concentrations of azide-gelatin induced larger hydrogel size after 7 days of incubation (Figure 3), which indicates that hydrogel swelling is promoted by the amount of gelatin. In contrast, the higher modification rate of azide-gelatin induced a smaller hydrogel size after 7 days of incubation (Figure 3). We reported that the turbidity of the solution of DBCO-PC-4armPEG crosslinker increased at temperatures above 30 °C [41], indicating polymer condensation due to hydrophobic interactions between DBCO–PC moieties. In general, hydrophobic groups induce shrinkage in hydrogels due to their hydrophobic interactions [56]. During higher modification rate of azide-gelatin, the concentration of counter DBCO moieties was high, which presumably reduced the hydrogel swelling and induced hydrogel shrinkage due to the hydrophobic properties of DBCO–PC moieties. Furthermore, the balanced azide-gelatin concentration and modification rate in PDH(24)-15, PDH(50)-30, PDH(50)-45, and PDH(75)-30 resulted in a size change of less than 5%.

While examining the hydrogel composition capable of HUVEC adhesion and growth, the lower modification rate of azide moieties and higher concentration of azide-gelatin solution induced greater adhesion and growth of HUVECs (Table 2). Gelatin was derived from the partial hydrolysis of native collagen, which has often been used for cell culture and it contains integrin-binding sites, such as the Arg-Gly-Asp motif for cell adhesion [57,58]. Based on the experimental results, it was indicated that the photodegradable hydrogels that were prepared at high azide-gelatin concentrations and low modification rate had enough integrin-binding sites and resulted in uniform HUVEC adhesion. HUVEC adhesion was nonuniform on the photodegradable hydrogels at high concentrations of azide-gelatin as they induced quick hydrogel formation at room temperature and resulted in nonhomogeneous crosslinking in the hydrogels.

We demonstrated the fabrication of linear and branched hollow structures with 25 µm × 25 µm in the Y × Z cross-section. It took about 1.3 and 2.7 h to fabricate the linear and branched hollow structures, respectively, with the fabrication condition of 0.7 µm of *ΔY*, 1.0 µm of *ΔZ*, and 750 µm/s of *V*_X_. Much more time is required for fabricating a complicated and larger hollow structure. This issue should be addressed by optimizing the laser scanning conditions.

In this study, we did not succeed in introducing the cells into the hollow structure when we observed the introduction of microspheres with a 9.9 µm diameter. To introduce cells into a hollow structure, we may presumably need to fabricate a structure larger than 25 µm × 25 µm because certain cells generally aggregate in the cell suspension. We think that the fabricated hollow structure should work as a scaffold for HUVEC adhesion and growth as investigated in this study and in our previous study [43]. Although fabrication of a larger hollow structure requires longer fabrication time, we would like to continue the work on the cell introduction and blood tissue engineering in the future.

## 5. Conclusions

In this study, we developed a novel method to fabricate the designed hollow structures in photodegradable hydrogels prepared in a microfluidic device in programmed manner. The hollow structures were fabricated in the hydrogels via multi-photon excitation process using an infrared femtosecond pulsed laser. Moreover, we found that the hydrogel composition with small size changes by less than 5% after 7 days incubation in PBS, and uniform adhesion and growth of HUVECs was observed. Using this hydrogel composition, we successfully fabricated perfusable linear and branched hollow structures in the photodegradable hydrogels prepared in the microfluidic device. 

## Figures and Tables

**Table 1:** Composition of the photodegradable hydrogels.

Type of the Photodegradable Hydrogel	Type of Azide-Gelatin	Concentration (mg/mL)	
		Azide-Gelatin	DBCO-PC-4arm PEG Crosslinker
PDH(15)-7.5	Azide-gelatin (15)	7.5	2.9
PDH(15)-15		15	5.9
PDH(15)-30		30	11.7
PDH(15)-45		45	17.6
PDH(15)-60		60	23.5
PDH(24)-7.5	Azide-gelatin (24)	7.5	4.9
PDH(24)-15		15	9.8
PDH(24)-30		30	19.5
PDH(24)-45		45	29.3
PDH(24)-60		60	39.1
PDH(50)-7.5	Azide-gelatin (50)	7.5	9.8
PDH(50)-15		15	19.5
PDH(50)-30		30	39.1
PDH(50)-45		45	58.6
PDH(50)-60		60	78.2
PDH(75)-7.5	Azide-gelatin (75)	7.5	14.7
PDH(75)-15		15	29.3
PDH(75)-30		30	58.6
PDH(75)-45		45	88.0
PDH(75)-60		60	117.3

**Table 2:** The effect of hydrogel composition on the size change of the photodegradable hydrogels on Day 7 from Day 0, and human umbilical vein endothelial cells (HUVEC) adhesion and growth. Data are presented as (size change)/(HUVEC adhesion and proliferation). Bold letters indicate the appropriate conditions for use in blood vessel tissue engineering.

Concentration of Azide-Gelatin Solutions (mg/mL)	Type of Azide-Gelatin			
	Azide-Gelatin (15)	Azide-Gelatin (24)	Azide-Gelatin (50)	Azide-Gelatin (75)
7.5	d	d	d	0.88/Partial
15	1.15/**Uniform**	**1.03** **/Uniform**	0.93/Partial	0.89/Partial
30	1.26/**Uniform**	1.10/**Uniform**	**0.96**/**Uniform**	**1.01** **/Uniform**
45	1.28/Nonuniform	1.09/Nonuniform	**1.03**/Nonuniform	i
60	1.18/Nonuniform	1.15/Nonuniform	i	i

d indicates the destruction of the photodegradable hydrogels when the hydrogels were retrieved from the silicon tubes. i indicates the insolubility of the DBCO-PC-4armPEG crosslinker to PBS.
